# Supplementary material for: The effectiveness of different attentional foci on the acquisition of sport-specific motor skills in healthy adults: a systematic review with network meta-analysis
Source: PeerJ. 2024 Aug 5;12:e17799. doi: 10.7717/peerj.17799 (PMC11308994; doi:10.7717/peerj.17799)

**Appendix**

Appendix A.1. Search string used in Medline.

# Search 
1 randomized controlled trial.pt. 
2 controlled clinical trial.pt. 
3 randomized.ab. 
4 placebo.ab. 
5 drug therapy.fs. 
6 randomly.ab. 
7 trial.ab. 
8 groups.ab. 
9 1 or 2 or 3 or 4 or 5 or 6 or 7 or 8 
10 sport*.mp. or exp Sports/ 
11 adult*.mp. 
12 exp Athletes/ or athlete*.mp. 
13 novice*.mp. 
14 expert*.mp. 
15 player*.mp. or exp Sports/ 
16 healthy.mp. 
17 beginner*.mp. [mp=title, book title, abstract, original title, name of substance word, 
subject heading word, floating sub-heading word, keyword heading word, organism 
supplementary concept word, protocol supplementary concept word, rare disease 
supplementary concept word, unique identifier, synonyms] 
18 learner.mp. 
19 participant*.mp. 
20 10 or 11 or 12 or 13 or 14 or 15 or 16 or 17 or 18 or 19 
21 acquisition.mp. 
22 accuracy.mp. 
23 speed.mp. 
24 velocity.mp. 
25 "skill acquisition".mp. 
26 "skill learning".mp. 
27 performance*.mp. 
28 exp Motor Skills/ or "motor performance".mp. 
29 complexity.mp. 
30 "movement quality".mp. 
31 "movement kinematics".mp. 
32 exp Kinetics/ or kinetic*.mp. 
33 "lower extremity kinematics".mp. 
34 power.mp. 
35 score*.mp. 
36 balance.mp. 
37 height*.mp. 
38 retention*.mp. 
39 transfer*.mp. 
40 dynamic balance task.mp.

41 landing forces.mp. 
42 landing mechanics.mp. 
43 movement preparation.mp. 
44 movement execution.mp. 
45 skill execution.mp. 
46 task execution.mp. 
47 golf.mp. 
48 21 or 22 or 23 or 24 or 25 or 26 or 27 or 28 or 29 or 30 or 31 or 32 or 33 or 34 or 35 
or 36 or 37 or 38 or 39 or 40 or 41 or 42 or 43 or 44 or 45 or 46 or 47 
49 "attentional focus".mp. 
50 "focus of attention".mp. 
51 "attentional foci".mp. 
52 "external focus".mp. 
53 "internal focus".mp. 
54 "combined focus".mp. 
55 "holistic focus".mp. 
56 "imagined external focus".mp. 
57 "switching focus".mp. 
58 "external foci".mp. [mp=title, book title, abstract, original title, name of substance 
word, subject heading word, floating sub-heading word, keyword heading word, organism 
supplementary concept word, protocol supplementary concept word, rare disease 
supplementary concept word, unique identifier, synonyms] 
59 internal foci.mp. [mp=title, book title, abstract, original title, name of substance 
word, subject heading word, floating sub-heading word, keyword heading word, organism 
supplementary concept word, protocol supplementary concept word, rare disease 
supplementary concept word, unique identifier, synonyms] 
60 increased focus.mp. 
61 attentional focus strategies.mp. 
62 combined strategy.mp. 
63 attentional switching.mp. 
64 OPTIMAL theory.mp. 
65 49 or 50 or 51 or 52 or 53 or 54 or 55 or 56 or 57 or 58 or 59 or 60 or 61 or 62 or 63 
or 64 
66 9 and 20 and 48 and 65

Appendix A.2. P-Scores post-acquisition testing

|  | **P-Score** |
| --- | --- |
| **Control** | 0.13 |
| **Internal focus of attention** | 0.21 |
| **Holistic focus** | 0.67 |
| **Switching focus** | 0.68 |
| **External focus of attention** | 0.81 |

Appendix A.3. Netleague table – post-acquisition testing

|  | **V1** | **V2** | **V3** | **V4** | **V5** |
| --- | --- | --- | --- | --- | --- |
| **1** | Control | -1.07 (-1.70; -0.44) | -0.92 (-2.22;  0.38) | 0.03 (-0.68;  0.73) | . |
| **2** | -0.99 (-1.57; -0.40) | External focus of attention | -0.08 (-1.36;  1.20) | 0.85 ( 0.45;  1.26) | -0.09 (-1.27;  1.09) |
| **3** | -0.81 (-1.93;  0.31) | 0.18 (-0.90;  1.26) | Holistic focus | 0.33 (-0.95;  1.61) | . |
| **4** | -0.11 (-0.71;  0.49) | 0.87 ( 0.47;  1.27) | 0.70 (-0.39;  1.78) | Internal focus of attention | -0.47 (-1.66;  0.72) |
| **5** | -0.83 (-2.00;  0.34) | 0.15 (-0.89;  1.20) | -0.02 (-1.50;  1.46) | -0.72 (-1.76;  0.33) | Switching focus |

NB: Effect estimates and 95%CI from pair-wise comparisons are presented in the upper right triangle. Network effect estimates are presented int the lower left triangle.

Appendix A.4. Heat plot – post-acquisition testing


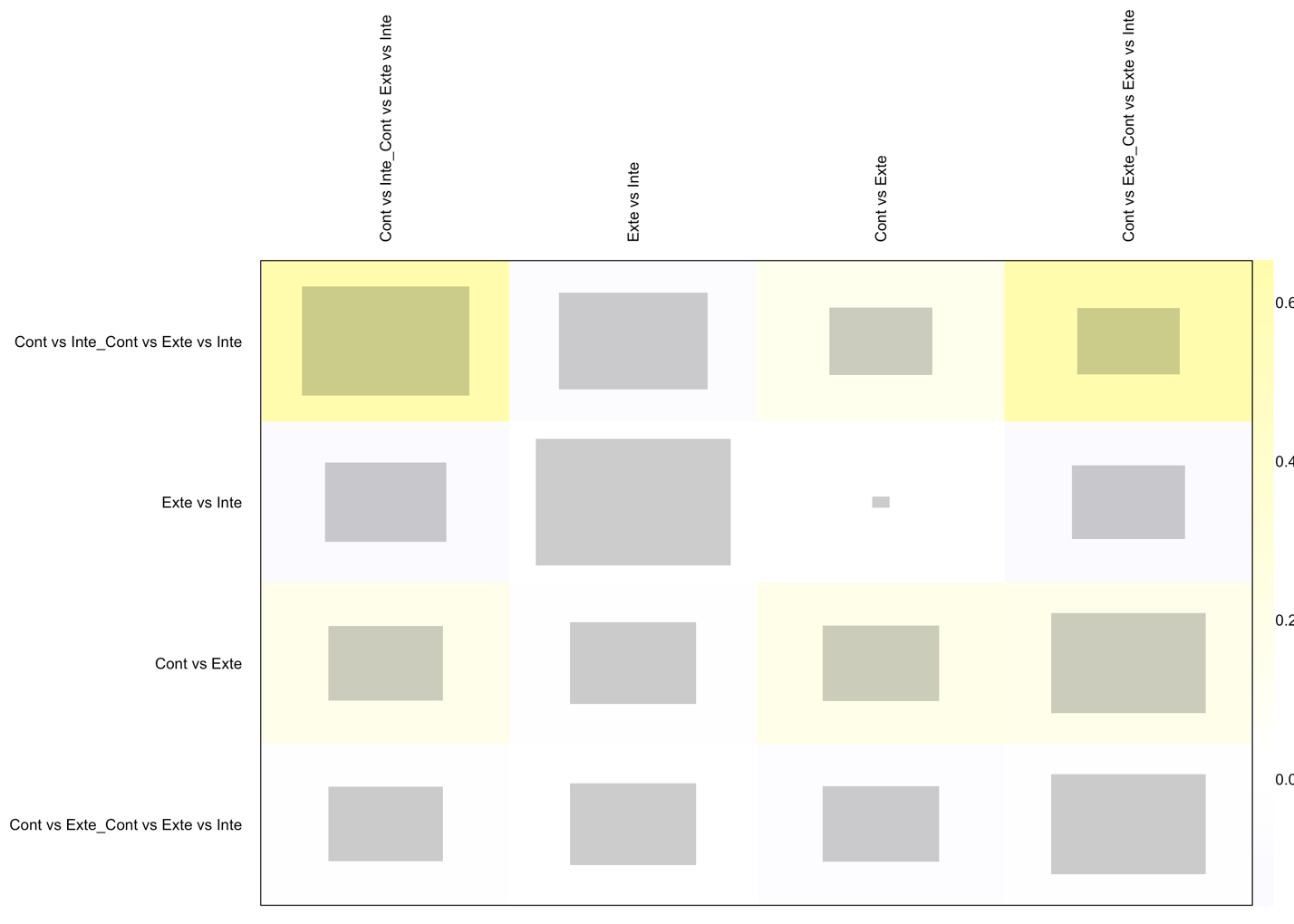


NB. Size of the boxes indicate the importance of a treatment comparison for the estimation of another treatment comparison. The colours indicate network inconsistency. Range is from blue (low inconsistency) to red (large inconsistency)

Appendix A.5. Funnel plot – post acquisition testing


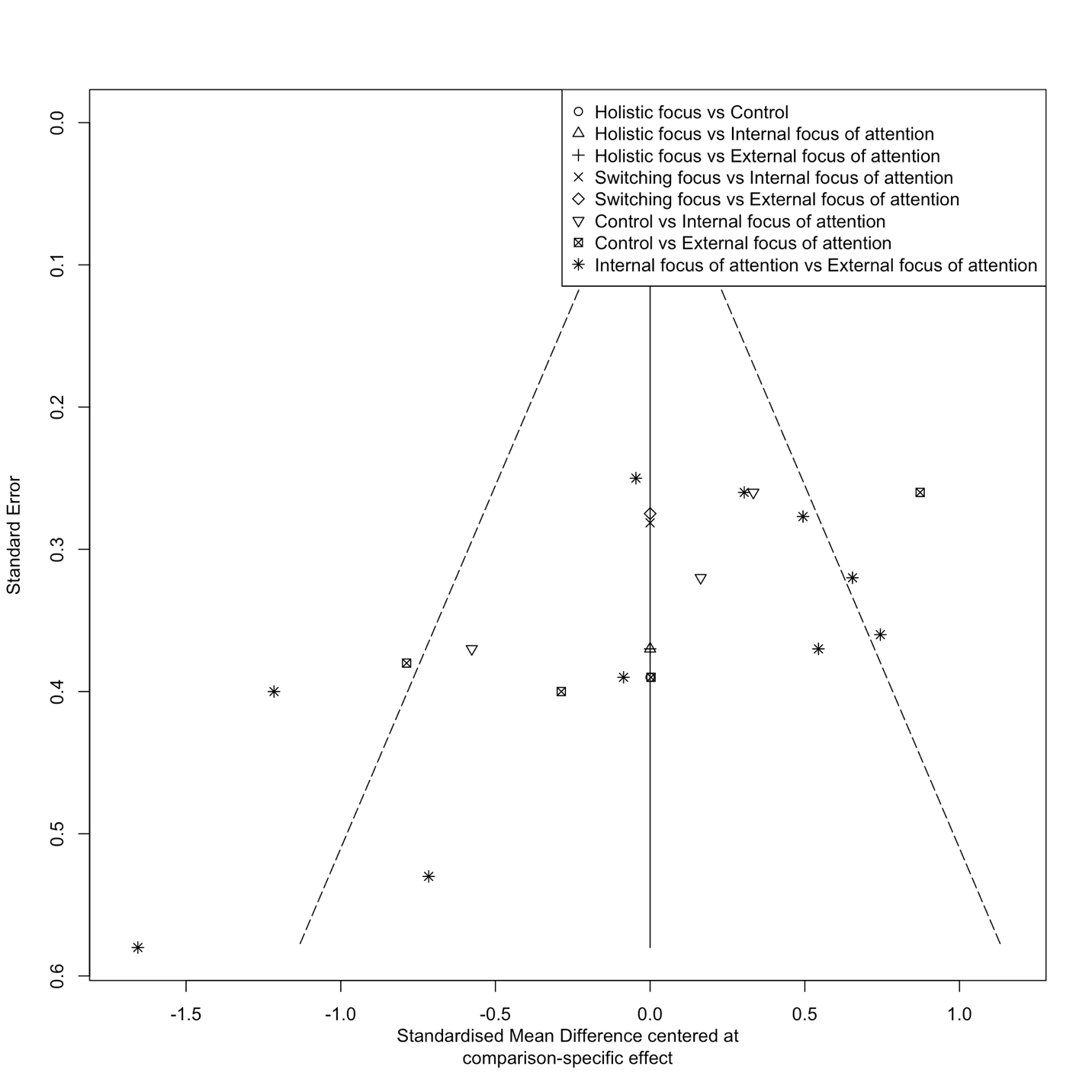


Appendix A.6. Netleague table – retention testing

|  | **V1** | **V2** | **V3** | **V4** | **V5** |
| --- | --- | --- | --- | --- | --- |
| **1** | Control | -0.20 (-1.00; 0.60) | . | 0.52 (-0.29; 1.33) | . |
| **2** | -0.12 (-0.84; 0.60) | External focus of attention | -0.21 (-1.16; 0.74) | 0.56 ( 0.20; 0.92) | -0.29 (-1.12; 0.53) |
| **3** | -0.30 (-1.39; 0.78) | -0.18 (-1.03; 0.66) | Holistic focus | 0.71 (-0.25; 1.68) | . |
| **4** | 0.44 (-0.28; 1.16) | 0.56 ( 0.20; 0.92) | 0.75 (-0.10; 1.60) | Internal focus of attention | -0.20 (-1.02; 0.63) |
| **5** | -0.09 (-1.08; 0.91) | 0.04 (-0.70; 0.77) | 0.22 (-0.88; 1.32) | -0.53 (-1.27; 0.21) | Switching focus |

NB: Effect estimates and 95%CI from pair-wise comparisons are presented in the upper right triangle. Network effect estimates are presented int the lower left triangle.

Appendix A.7. Net splitting – retention testing

Appendix A.8. Comparison adjusted funnel plot – retention testing

Appendix A.9. Netleague table – transfer testing

|  | **V1** | **V2** | **V3** | **V4** |
| --- | --- | --- | --- | --- |
| **1** | External focus of attention | -0.91 (-1.66; -0.15) | 0.37 ( 0.03;  0.70) | 0.04 (-0.50;  0.57) |
| **2** | -0.82 (-1.51; -0.12) | Holistic focus | 1.06 ( 0.29;  1.84) | . |
| **3** | 0.35 ( 0.02;  0.68) | 1.16 ( 0.47;  1.86) | Internal focus of attention | -0.07 (-0.61;  0.48) |
| **4** | 0.16 (-0.34;  0.65) | 0.97 ( 0.15;  1.79) | -0.19 (-0.69;  0.31) | Switching focus |

NB: Effect estimates and 95%CI from pair-wise comparisons are presented in the upper right triangle. Network effect estimates are presented int the lower left triangle.

Appendix A.10. Net splitting – transfer testing

Appendix A.11. Sensitivity analysis post-acquisition

In this analysis we excluded the study of Wulf et al (1999) in order to investigate the influence on inconsistency of this study.


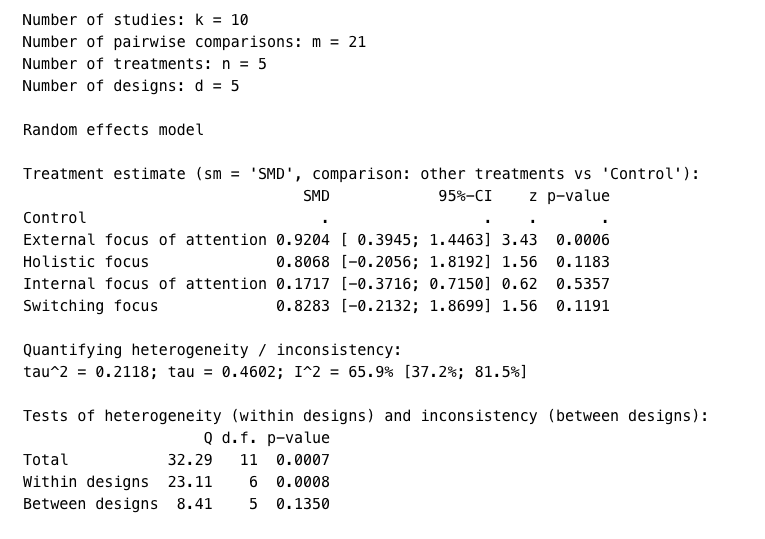

Supplement: Supplemental Information 2 [file peerj-12-17799-s002.docx]
