# Supplementary material for: The effectiveness of different attentional foci on the acquisition of sport-specific motor skills in healthy adults: a systematic review with network meta-analysis
Source: PeerJ. 2024 Aug 5;12:e17799. doi: 10.7717/peerj.17799 (PMC11308994; doi:10.7717/peerj.17799)
Supplement: Supplemental Information 7 [file peerj-12-17799-s007.docx]

## The rationale for conducting the systematic review / meta-analysis;

The acquisition of motor skills is a key element in many sports. A motor learning principle, which is frequently used in practice to increase skill acquisition is the application of different attentional foci. This motor learning principle was originally investigated by comparing an internal and an external focus of attention (Wulf et al., 2001).

Several randomised controlled trials have reported the effectiveness of different attentional foci on the acquisition of sports-related motor skills. In addition to internal and external foci of attention, these studies propose other foci of attention (such as a holistic focus (Abedanzadeh et al., 2022)) or modifications of an existing focus of attention (such as an imagined external focus (Yamada et al., 2021)).

## 2) The contribution that it makes to knowledge in light of previously published related reports, including other meta-analyses and systematic reviews.

Current systematic reviews (Chua et al., 2021; Makaruk et al., 2020) analyse the effectiveness of attentional foci using traditional pairwise meta-analyses. This limits the analyses to the comparisons: external versus internal, external versus control and internal versus control. Therefore, other proposed attentional foci in primary studies such as an “holistic” or a “switching” focus of attention are currently not analysed with a meta-analysis.

In our study, we used a network meta-analysis, which allowed us to extend the analyses to include other attentional focus interventions reported in primary trials. Furthermore, based on the analyses, we were able to create a treatment ranking of the included attentional foci. The results of this systematic review provide information with important implications for further research and practice. The main findings of the systematic review are:

- An external focus showed greater effectiveness compared to an internal focus of attention on acquisition of motor tasks in healthy adults.
- Several different attentional foci interventions with promising effectiveness were identified such as a holistic focus of attention or an imagined focus of attention.
- Despite a large body of evidence, there is still a lack of larger studies investigating the effectiveness of different attentional foci.

**References:**

Abedanzadeh, R., Becker, K., & Mousavi, S. M. R. (2022). Both a holistic and external focus of attention enhance the learning of a badminton short serve. *Psychological Research*, *86*(1), 141–149.

Chua, L.-K., Jimenez-Diaz, J., Lewthwaite, R., Kim, T., & Wulf, G. (2021). Superiority of external attentional focus for motor performance and learning: Systematic reviews and meta-analyses. *Psychological Bulletin*, *147*(6), 618. https://pubmed.ncbi.nlm.nih.gov/34843301/

Makaruk, H., Starzak, M., & Porter, J. M. (2020). Influence of attentional manipulation on jumping performance: A systematic review and meta-analysis. *Journal of Human Kinetics*, *75*(1), 65–75. https://pubmed.ncbi.nlm.nih.gov/33312295/

Yamada, M., Raisbeck, L. D., & Porter, J. M. (2021). The Effects of Using Imagery to Elicit an External Focus of Attention. *Research Quarterly for Exercise and Sport*, *92*(3), 559–565. https://doi.org/10.1080/02701367.2020.1733455
